# Upcycling Sugar Cane Biomass into 2G Sugars and Lignin-Derived Biochars for Preparing Carbon-Based Electrodes

**DOI:** 10.1021/acsomega.5c08482

**Published:** 2025-10-30

**Authors:** Lucas Ramos, Talita M. Lacerda, André Ferraz, Mariusz Grabda, Sylwia Oleszek, Hideyuki Horino, Izabela Rzeznicka, Anuj Kumar Chandel

**Affiliations:** † Renewable Carbon and Biology Systems (ReCABS) Laboratory, Department of Biotechnology, 119119 Engineering School of Lorena, University of São Paulo (EEL-USP), Lorena, São Paulo 12602-810, Brazil; ‡ 496994 Shibaura Institute of Technology Research Laboratories, Saitama 337-8570, Japan; § Department of Environmental Engineering, Graduate School of Engineering, 12918 Kyoto University, Kyoto 615-8540, Japan; ∥ Research Management Center, 13101 Tohoku University, Sendai 982-8577, Japan; ⊥ College of Engineering, Shibaura Institute of Technology, Saitama 337-8570, Japan; ∞ Department of Biotechnology, 119119 Engineering School of Lorena, University of São Paulo (EEL-USP), Lorena, São Paulo 12602-810, Brazil

## Abstract

Converting lignin into specialty and bulk chemicals enhances both the economic viability and the sustainability of biorefineries. Here, we present an integrated approach to produce monosaccharides, lignin, and lignin-derived biochars from two underutilized agricultural byproducts: sugar cane bagasse and sugar cane straw. Modified kraft and soda pulping yielded digestible pulps, which were readily hydrolyzed into monosaccharides with glucan conversions ranging from 76% to 96%. The corresponding pretreatment liquors provided lignins, which were pyrolyzed to obtain biochars. These biochars were blended with a binder to prepare biochar inks, which were deposited onto electrodes to create functional electrodes. SEM-EDX characterization revealed a higher silicate content in straw-derived biochars and increased sulfur levels in kraft-derived biochars. Such compositional differences influenced electrochemical stability and catalytic activity toward oxygen evolution (OER) and oxygen reduction (ORR) reactions. Bagasse-derived electrodes exhibited modest ORR currents, with onset potentials of 0.82 V (soda) and 0.76 V (kraft). In contrast, straw-derived electrodes displayed pronounced electrochemical instability in alkaline media, limiting their use as catalytic carbons for metal–air batteries. Nonetheless, their higher conductivity suggests potential as conductive additives in insertion-type battery electrodes. Overall, this work illustrates integrated strategies for valorizing agricultural byproducts, expanding the range of applications for lignin-derived materials within biorefinery processes.

## Graphical abstract

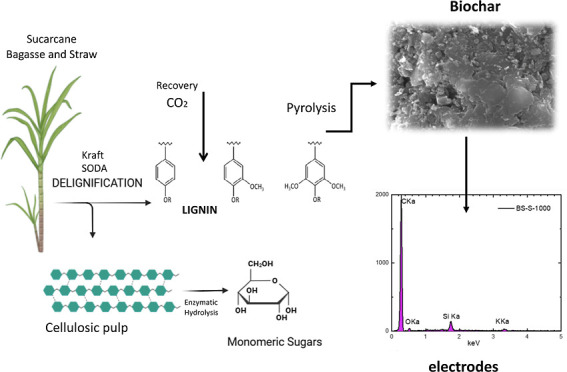

## Introduction

Lignocellulosic biomass has gained considerable attention as a feedstock for producing bioenergy and biobased products, particularly those derived from agricultural wastes and energy crops (Armah et al., 2019 ^1^). Current biorefineries processing lignocellulosic materials primarily focus on converting polysaccharide fractions into biofuels and renewable chemicals, whereas lignin is commonly combusted on-site for heat and power generation. This practice is largely driven by lignin’s inherent structural heterogeneity, chemical instability, and the limited availability of economically viable valorization routes (Dias et al., 2013;^2^ Martins et al., 2024 ^3^). Ongoing research and development in lignin valorization have revealed significant potential for applications such as lignin-based hydrogels, surfactants, adhesives, three-dimensional printing materials, technical carbons, and fine chemicals (Sethupathy et al., 2022 ^4^). Furthermore, converting lignin into specialty and bulk chemicals can improve the economic viability of biorefineries, offering a more favorable trade-off for sugar cane mills and second-generation (2G) ethanol production facilities. To maximize commercial benefits, it is advisable to allocate a portion of the extracted lignin for the production of high-value products such as adhesives, carbon inks, dispersants, and surfactants (Boschetti et al., 2019 ^5^). However, combusting a substantial share of lignin remains essential for recovering inorganics in the kraft and soda processes as well as for generating steam and energy. In the sugar cane industry, steam produced from lignin or biomass combustion is critical for meeting the operational demands of ethanol production.

The market outlook for lignin and lignin-based products is promising, with the global market valued at US$ 106.1 million in 2022 and projected to reach US$ 1554 million by 2029, representing a compound annual growth rate of 5.0% (Market Growth Reports, 2025 ^6^).

To achieve both profitability and sustainability in plant biomass processing, all biomass fractions should be utilized to produce a range of value-added products. Consequently, effective biorefining of plant biomass requires pretreatment steps, followed by the conversion of the resulting fractions. Among the various options, alkaline pretreatment processes are among the simplest and have been widely implemented at an industrial scale, particularly in the well-established pulping industry (Sewsynker-Sukai et al., 2020 ^7^). In this process, lignin is separated from the plant biomass, resulting in two main streams: a lignin-rich fraction contained in the pulping liquor and a cellulosic pulp. While cellulosic pulps derived from wood resources have a well-established and sizable market (Klein and Luna, 2023 ^8^), pulps obtained from grass biomasses typically yield a polysaccharide-enriched fraction with poor papermaking quality (Danielewicz et al., 2021).^9^ Utilizing this pulp for monosaccharide production—and potentially for second-generation (2G) ethanol—may represent a more suitable and value-added application. As for the lignin stream, its use in polymeric form presents several challenges, primarily due to the highly variable and heterogeneous structure of lignin, which depends on both the type of plant biomass and the extraction method employed (Kent et al., 2018 ^10^).

It is worth emphasizing that studies reported in the scientific literature concerning lignin conversion remain exceedingly limited, particularly those addressing its potential application in the development of printed electrodes. This evident gap underscores a promising, yet underexplored, research avenue with substantial potential for technological innovation.

This study reports an integrated biorefinery strategy for the valorization of sugar cane bagasse and straw, two abundant yet underutilized lignocellulosic residues characterized by limited suitability for high-quality papermaking due to their fiber properties. To address their low economic value in conventional applications, we employed established industrial kraft and soda pretreatment processes to enable scalable and efficient fractionation, producing monosaccharides, lignin, and lignin-derived biochar. This approach leverages existing industrial infrastructure and enhances the valorization of both carbohydrate and lignin fractions, aligning with the principles of sustainability and circular bioeconomy development.

The production of lignin-derived biochar with tailored properties was pursued for the subsequent formulation of catalytic inks for the printed electrodes. Extracted lignin samples underwent high-temperature pyrolysis to generate biochar, which was characterized by Raman spectroscopy and scanning electron microscopy (SEM) coupled with energy-dispersive X-ray spectroscopy (SEM-EDX). The resulting biochar powders were used to prepare electrode inks, which were then evaluated for their electrochemical performance in alkaline solutions.

## Materials and Methods

### Materials

Sugar cane bagasse and straw were obtained from the 2022–2023 harvesting season and kindly provided by a sugar cane mill located in Descalvado, São Paulo, Brazil. The bagasse was air-dried and stored under dry conditions for subsequent processing and chemical characterization. The sugar cane straw was initially blended with water to remove excess soil and other impurities. The washed straw was retained using a 20-mesh nylon screen during centrifugation, while the soil residues were discarded with washing water. Cellic CTec3 used in enzymatic hydrolysis experiments was provided by Novozymes, Latin America (Brazil). *N*-methyl-2-pyrrolidine (NMP, 99.5%) was purchased from FUJIFIM Wako Pure Chemicals Co. Polyvinylidene fluoride (PVDF) used as a binder was obtained from Kureha, Japan. Acetylene black (99.99%) was purchased from Strem Chemicals. A 1 mol/L KOH solution was purchased from Sigma-Aldrich and used without further purification. A glassy carbon-rotating disk electrode (GC-RDE) with a diameter of 3 mm, a Hg/HgO (1 mol/L NaOH) electrode, and a platinum rod were purchased from EC Frontier Inc., Tokyo, Japan. The surface of the GC-RDE was polished using a 0.05 μm alumina slurry and washed with hydrochloric acid and ethanol under sonication prior to performing electrochemical experiments. All other reagents were of analytical grade.

### Biomass Pretreatment and Lignin Recovery

Sugar cane bagasse and straw were pretreated using either the kraft or soda process. For the kraft pretreatment, previously prepared solutions of NaOH (3.5 mol/L) and Na_2_S (0.6 mol/L) were mixed to achieve the desired active alkali and sulfidity levels for each experimental condition. Four different pretreatment runs, using varying active alkali loadings, were carried out simultaneously using four 1-L reactors operated in an AU/E-20 REGMED digester, rotating at four cycles per minute. The active alkali loads used were 9, 11, 13, and 15%, calculated as the combined mass of NaOH and Na_2_S in the reactor, expressed as grams per 100 g of lignocellulosic biomass. Sulfidity, defined as the ratio of Na_2_S to active alkali, was fixed at 25% for all experiments. The biomass-to-liquor ratio was maintained at 1:10 (w/v). Pretreatment was conducted at a target temperature of 170 °C, which was reached after a 1 h heating ramp. The maximum temperature (170 °C) was then held constant for 3 h (Gonçalves et al., 2005; Mamaye et al., 2022).^11,12^ Following the reaction period, the reactors were cooled in a water bath until the temperature reached 80 °C. The solid and liquid phases resulting from the pretreatment were separated by centrifugation through a 20-mesh screen. The liquid fraction was immediately flushed with a nitrogen stream and stored at 4 °C in plastic bottles until further use. The digested solids were washed with water until the rinsewater reached neutral pH (pH 7), then air-dried, and stored at 4 °C in plastic bags for subsequent analysis.

Soda cooking experiments were conducted under similar conditions but without the addition of sodium sulfide (0% sulfidity).

Untreated and pretreated biomass samples were characterized according to chemical compositions following procedures previously described (Ferraz et al., 2000 ^13^). Shortly, samples were hydrolyzed with sulfuric acid in a two-step procedure where the residual solids were determined gravimetrically as klason insoluble lignin. The soluble fraction containing monosaccharides were analyzed by high-performance liquid chromatography (HPLC) using an Aminex HPX-87H column (Bio-Rad, Hercules, CA, USA) and a Waters 1515 isocratic HPLC pump, and Waters 2414 refractive index detector with 5 mM H_2_SO_4_ eluent and 0.6 mL/min eluent flow.

### Enzymatic Hydrolysis of the Cellulosic Pulps

Enzymatic hydrolysis of cellulosic pulps was carried out using a commercial cellulase preparation, Cellic CTec3 (Novozymes, Latin America, Brazil), at a loading of 10 FPU/g of pretreated biomass. The hydrolysis was conducted at a temperature of 50 °C and stirring speed of 200 rpm for 72 h. Glucose and xylose concentrations in the liquid fractions were determined by HPLC using an Aminex HPX-87H column (Bio-Rad, Hercules, CA, USA) and a Waters 1515 isocratic HPLC pump, and Waters 2414 refractive index detector with 5 mM H_2_SO_4_ eluent and 0.6 mL/min eluent flow as described by Várnai et al. (2014).^14^

### Lignin Recovery from the Pretreatment Liquors

Pretreatment liquors were acidified by bubbling carbon dioxide at a flow rate of 9.9 L/h for 5 min. The temperature was maintained at 70 °C throughout the acidification process (Tomani, 2009; Bertaud et al., 2023).^15,16^ During this step, a portion of the lignin present in the liquor precipitated and was recovered by centrifugation (20 min, 4500 rpm). The resulting solid fraction was washed twice with a 2% (w/v) aqueous sulfuric acid solution and once with distilled water to remove residual acid. The washed solids were then dried at 60 °C in a vented oven. Lignin concentrations in the pretreatment liquors and in the supernatant obtained after centrifugation were determined by ultraviolet spectroscopy at 280 nm. Samples were diluted in 10 mM NaOH to yield absorbance values within the range of 0.7–1.2. Absorbance readings were converted to lignin concentrations using an estimated absorptivity of 20 L/g·cm according to Tamminen and Hortling (1999).^17^

Elemental analysis (CHNO) of the lignin solid fraction was carried out using a CHN Analyzer Series II (PerkinElmer, USA), and sulfur contents were estimated with inductively coupled plasma optical emission spectrometry (Oliveira et al., 2015)^18^ at the Instrumental Analytical Center of the University of São Paulo.

### Pyrolysis-Gas Chromatography/Mass Spectrometry (Py-GC/MS) Evaluation of the Lignin Samples’ Composition

Pyrolysis experiments were performed using a commercial pyrolyzer (Py, EGA/Py-3030D, Frontier Laboratories, Inc., Japan), combined with a gas chromatograph and mass spectrometer (GC/MS-QP2020NX, Shimadzu Corp., Japan). A sample (0.21–0.26 mg) was introduced into a quartz cup and covered with quartz wool to prevent scattering. The sample was pyrolyzed in a single-shot mode at 600 °C for 20 s under the stream of high-purity helium (He, 99.999%, at the flow rate of 1 cm^3^ min^–1^). The Py unit sits on top of a standard splitless inlet on the GC/MS unit equipped with an Ultra ALLOY-5 capillary column (30 m × 0.25 mm × 0.25 mm, length, inner diameter, and thickness, Frontier Laboratories, Japan). The interface between Py and GC was kept at 300 °C to avoid condensation of the vaporized compounds. The GC inlet was kept at 250 °C, and a split ratio of 50:1 (GC/MS) was used. The temperature of the GC oven was raised from 50 to 300 °C using a 10 °C/min linear heating rate and kept at 300 °C for 25 min. The mass spectra were recorded under 70 eV electron ionization conditions with the *m*/*z* from 29 to 700 amu. The pyrolysis products were identified based on the reported literature and by comparing a mass spectrum with the mass spectrum available at the NIST library. The analysis was performed twice for each sample.

### Biochar Production and Characterization

#### Biochar Production

Biochar powders were produced by thermal conversion of lignin material under pyrolytic conditions. About 1.0–1.5 mg of the lignin material was loaded in a high-purity alumina crucible and placed in the center of a horizontal tubular ceramic furnace (ARF-30KC, Asahi-Rika, Japan) equipped with a precise temperature controller (AGC-1P, Asahi-Rika, Japan). The furnace was heated from 20 °C up to 1000 °C with the heating rate of 10 °C min^–1^ under nitrogen at the flow rate of 100 cm^3^ min^–1^. The heating program included three steps of isothermal heating of the lignin at 180, 350, and 1000 °C for 1 h to obtain a uniformly pyrolized product. The total conversion time of the lignin with the above procedure was 4 h 38 min. A soak time of 1 h in N_2_, 100 cm^3^ min^–1^ was applied before initialization of the heating and during the cooling of the product to the room temperature to ensure highly inert conditions during production. After pyrolysis, biochar powders were collected from the cup and stored under dry conditions for further characterization. Characterization was performed on biochar powders sieved through 38 μm test sieves.

#### Scanning Electron Microscopy Energy-Dispersive X-ray Spectroscopy

Sieved biochar powders were deposited on conductive carbon tape and attached to a sample holder made of stainless steel. No conductive metal layer was used for imaging. The images were obtained using a field emission SEM unit (FE-SEM7100F, Jeol, Japan) equipped with an EDX detector. The images were acquired at a 15 keV accelerating voltage, and EDX spectra were acquired at a current of 11 mA from the 5 × 5 μm area of the sample.

#### Raman Spectroscopy

Raman spectra were acquired using a LabRAM HR Evolution confocal Raman microscope (HORIBA Scientific, Japan) equipped with a 532 nm diode laser and a 10× objective lens. Spectra were recorded using 600 gr/mm gratings, giving spectral resolution of 1.67 cm^–1^. Each spectrum was acquired for 10 s, and spectra were accumulated three times. A multichannel air-cooled (−70 °C) charge-coupled device detector (Syncerity, Horiba, Japan) was used as a detector. Before spectra acquisition, the *x*-axis was calibrated using the crystalline silicon line at 520.7 cm^–1^. A sample was prepared by mixing 3 mg of sieved carbon powder with 100 mg of moisture-free KBr powder (FT-IR grade, ≥99% trace metals basis, Sigma-Aldrich). The powder mixture was pelletized using a tablet press and used in this form for the acquisition of Raman spectra.

### Biochar Ink and Electrode Preparations

Biochar ink was prepared by mixing sieved biochar powder with a PVDF binder dissolved in NMP solution (2% (w/w)). The ratio of PVDF to carbon was (1:23). The mixture was stirred at 300 rpm for 1 h, and 1 μL of it was deposited on a clean glassy carbon electrode (GCE). The ink-coated electrode was dried in air for 2–6 h and then under vacuum at 80 °C for 6 h.

### Electrochemical Tests

Electrochemical tests were carried out in a three-electrode glass system using an automated polarization system (HZ-7000, Hokuto Denko Co., Tokyo, Japan). A GCE with a diameter of 3 mm coated with biochar ink was used as the working electrode. A Hg/HgO (1 M KOH) electrode was used as a reference electrode and Pt wire as a counter electrode (Putra et al., 2022 ^19^).

Working electrodes were investigated by linear sweep voltammetry (LSV) in a 1 M KOH solution under nitrogen- or oxygen-saturated conditions. The potential recorded with the Hg/HgO reference electrode was converted to the reversible hydrogen electrode using the Nernst equation, as expressed in eq 1.

1 $$E_{RHE}=E_{Hg/HgO\left(1\;M\;NaOH\right)}+E_{Hg/HgO\left(1\;M\;NaOH\right)}^{{}^{\circ}}+0.059\;\Delta pH$$

where *E*_Hg/HgO (1 M NaOH)_ is the recorded potential measured using the reference electrode; *E*_Hg/HgO (1 M NaOH)_^°^ is the standard potential of the Hg/HgO redox couple in 1 M NaOH (0.118 V); and ΔpH indicates the pH difference of the working solution with respect to the conditions applied in the normal hydrogen electrode (in this study, ΔpH = 14).

## Results and Discussion

### Process Yield and Chemical Composition of Pulps Prepared by Kraft and Soda Processing of Sugar Cane Bagasse and Sugar Cane Straw

Sugar cane bagasse and straw are abundant byproducts of the well-established sugar cane industry. Although they are primarily used for energy production, a portion of these lignocellulosic materials can be utilized to generate new products, enhancing biorefinery profitability and sustainability (Mujtaba et al., 2023).^20^ In this study, these materials were processed using traditional kraft and soda pulping methods to produce a digestible pulp suitable for monosaccharide production as well as lignin, which was converted into biochar and subsequently transformed into catalytic ink for the fabrication of printed electrodes. Table 1 summarizes the processing yield and chemical composition of untreated and treated materials. Mass balance for biomass components was calculated from the solids’ yield recorded from each pretreatment and the corresponding chemical compositions of prepared solids, considering that the yield was accounted for at each step to calculate the mass balance. Both alkaline processes are known for their selective lignin removal, resulting in a lignin-rich liquor and a solid pulp with reduced lignin content (Danielewicz et al., 2021; Ascencio et al., 2025).^9,21^ For both substrates, the kraft process was more efficient than the soda process in delignification due to the presence of hydrosulfide ions in the pulping liquor (Fearon et al., 2020).^22^ Sugar cane straw exhibited greater resistance to delignification compared to sugar cane bagasse, as its lignin content decreased to a lesser extent in both processes. As expected, higher active alkali loads in the processes enhanced the lignin removal for both substrates. The partial removal of hemicellulose was also proportional to the alkali load, while glucan remained largely preserved, even at the highest alkali concentrations. Deacetylation of hemicelluloses was also a major reaction due to the high alkali concentration and elevated cooking temperature.

**Table 1:** Yield of Pretreated Solids, Chemical Composition, and Mass Balance for Sugar Cane Bagasse and Straw Components after Pretreatment under Modified Kraft Pulping and Soda Pulping

biomass sample	pretreatment and active alkali load (%, w/w)	yield of pretreated solids (%)	chemical composition of biomass sample (g/100 g biomass sample)					mass balance of biomass components (g/100 g of original biomass sample)				
			lignin	glucan	xylan	arabinosyl	acetyl	lignin	glucan	xylan	arabinosyl	acetyl
Nontreated Sugar Cane Bagasse^a^												
		100	21.9 ± 0.4	40.7 ± 0.4	21.3 ± 0.1	1.9 ± 0.1	3.3 ± 0.1	21.9	40.7	21.3	1.9	3.3
Pretreated Sugar Cane Bagasse												
	kraft 9%	63	13.0 ± 0.2	57.5 ± 1.8	22.7 ± 0.6	1.8 ± 0.1	0.5 ± 0.0	8.2	36.3	14.4	1.1	0.3
	kraft 11%	61	10.0 ± 0.1	59.1 ± 0.6	23.9 ± 0.3	1.8 ± 0.1	0.6 ± 0.1	6.2	36.6	14.8	1.1	0.4
	kraft 13%	58	9.4 ± 0.6	58.4 ± 1.4	23.8 ± 0.5	1.8 ± 0.1	0.5 ± 0.1	5.5	33.9	13.8	1.0	0.3
	kraft 15%	57	5.1 ± 0.2	60.9 ± 0.9	25.2 ± 0.3	1.9 ± 0.1	0.5 ± 0.1	2.9	34.8	14.4	1.1	0.3
	soda 9%	68	14.9 ± 0.2	54.7 ± 1.6	20.8 ± 1.3	1.7 ± 0.1	0.2 ± 0.1	10.1	36.9	14.1	1.2	0.1
	soda 11%	65	13.7 ± 0.7	56.4 ± 1.5	21.9 ± 0.4	1.8 ± 0.1	0.2 ± 0.0	8.9	36.6	14.2	1.1	0.1
	soda 13%	62	11.6 ± 0.4	57.6 ± 1.0	20.9 ± 1.4	1.7 ± 0.1	0.2 ± 0.0	7.1	35.5	12.9	1.0	0.1
	soda 15%	57	7.2 ± 0.4	59.7 ± 1.4	24.4 ± 1.4	1.8 ± 0.1	0.2 ± 0.0	4.1	34.1	13.9	1.0	0.1
Nontreated Sugar Cane Straw^a^												
		100	25.8 ± 0.9	33.3 ± 0.9	19.5 ± 0.6	2.6 ± 0.1	2.1 ± 0.2	25.8	33.3	19.5	2.6	2.1
Pretreated Sugar Cane Straw												
	kraft 9%	51	18.5 ± 0.1	56.6 ± 0.5	19.2 ± 0.1	1.9 ± 0.1	0.6 ± 0.1	9.4	28.6	9.7	1.0	0.1
	kraft 11%	49	14.6 ± 2.0	57.4 ± 3.5	18.9 ± 0.8	2.0 ± 0.2	0.2 ± 0.1	7.1	27.8	9.2	1.0	0.1
	kraft 13%	48	12.7 ± 1.8	57.9 ± 0.7	20.5 ± 0.3	2.2 ± 0.1	0.6 ± 0.1	6.1	27.8	9.9	1.1	0.1
	kraft 15%	43	8.9 ± 3.4	59.8 ± 3.0	20.1 ± 1.8	2.2 ± 0.3	0.2 ± 0.1	3.8	25.5	8.6	0.9	0.1
	soda 9%	59	21.4 ± 0.7	53.4 ± 1.4	17.3 ± 0.5	1.7 ± 0.1	0.1 ± 0.1	12.5	31.4	10.2	1.0	0.1
	soda 11%	53	19.5 ± 0.7	54.1 ± 2.4	15.3 ± 0.7	1.5 ± 0.1	0.1 ± 0.1	10.2	28.4	8.0	0.8	0.1
	soda 13%	48	18.9 ± 1.5	58.2 ± 1.3	16.4 ± 0.4	1.5 ± 0.1	0.1 ± 0.1	9.1	28.0	7.9	0.7	0.1
	soda 15%	49	11.9 ± 0.7	60.1 ± 0.5	17.7 ± 0.2	1.7 ± 0.1	0.2 ± 0.1	5.9	29.6	8.7	0.8	0.1

a Extractives and ash contents in nontreated sugar cane bagasse were 2.0 ± 0.2% and 1.5 ± 0.2%, respectively. In sugar cane straw, these values were 2.9 ± 0.1% and 4.8 ± 0.1%, respectively.

Kraft and soda pulps from sugar cane bagasse have been industrially produced worldwide; however, their papermaking quality is relatively low (Mboowa, 2024).^23^ In the current biorefinery approach, we focused on evaluating the conversion of these pulps into monosaccharides through enzymatic hydrolysis using commercial cellulases. More than 70% (ranging from 76% to 96%) of the polysaccharides in pretreated sugar cane bagasse and straw were converted into monosaccharides, even after pretreatment with the lowest active alkali loads (Figure 1). Data in Table 1 show that lignin removal during pretreatment exceeded 50% even at the lowest alkali loads, which is the primary factor contributing to the high digestibility of the pretreated samples. For instance, lignin removal from sugar cane bagasse treated with 9% active alkali reached 62% and 54% in the kraft and soda processes, respectively. For sugar cane straw, these values were 63% and 51%, respectively. It is well established that lignin removal of approximately 50% during biomass pretreatment is sufficient to produce highly digestible materials (Ramos et al., 2021; Chourasia et al., 2021; Siqueira et al., 2013),^24–26^ which was confirmed by our current experiments. A comparison of kraft-pretreated samples at increasing alkali loads indicates that sugar cane bagasse was more digestible than sugar cane straw, achieving over 90% glucan and xylan conversions at 11% active alkali. In contrast, a similar digestibility was observed in sugar cane straw only at the highest alkali loads used in the pretreatment (15%). A similar trend was observed for soda-pretreated samples, though polysaccharide conversion levels were approximately 5% lower than those obtained with the kraft pretreatment.

**Figure 1:**
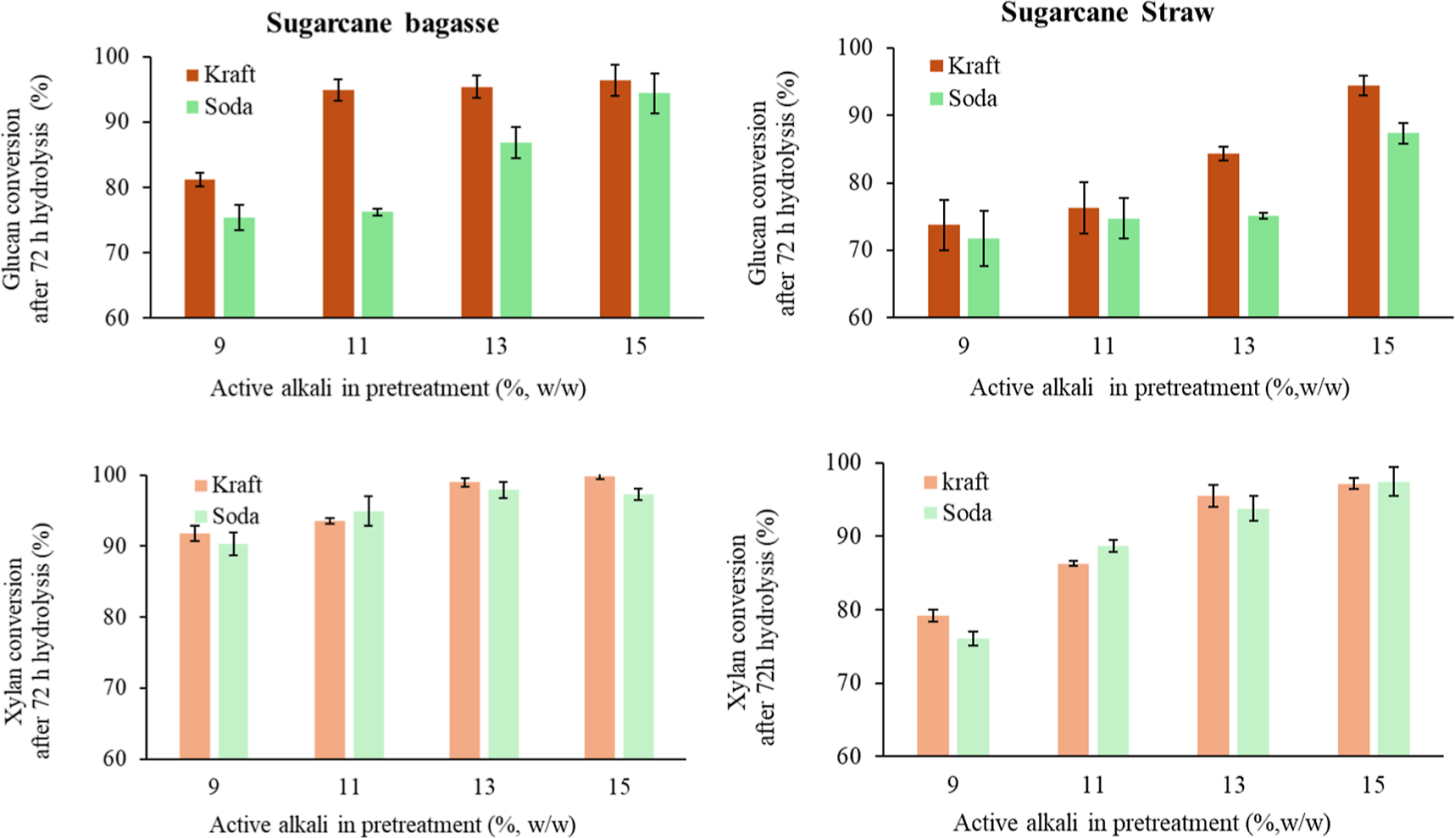
Glucan and xylan conversions after 72 h of enzymatic hydrolysis of sugar cane bagasse and straw pretreated by kraft and soda processes.

### Lignin Recovery from Pretreatment Liquors

Kraft and soda pulping processes have well-established recovery systems, in which pulping liquors are concentrated and combusted in recovery boilers. This enables the sustainable recycling of inorganic chemicals used in the processes while simultaneously generating process steam and electrical energy (Dias et al., 2013; Bertaud et al., 2023; Martins et al., 2024).^2,3,16^ A growing practice in these industries involves recovering a portion of the lignin from the liquor prior to combustion with the dual purpose of debottlenecking recovery boilers and obtaining a commercially valuable lignin byproduct. This step is typically carried out by acidifying the pulping liquor with CO_2_, followed by the recovery of the precipitated lignin, in a process known as LignoBoost (Tomani, 2009; Bertaud et al., 2023).^15,16^ In the present study, LignoBoost was applied to recover a fraction of the lignin from bagasse and straw pretreatment liquors (Tables S1 and S2). The pH of the liquors ranged from 7.8 to 10.2, depending on the original active alkali load used during cooking. A general trend observed was that sugar cane straw consumed more active alkali than bagasse. After acidification with CO_2_, the liquor pH decreased to between 6.9 and 8.5, promoting lignin precipitation. Lignin concentrations in the pretreatment liquors before and after CO_2_ acidification are presented in Figure 2. The proportion of lignin precipitated from the black liquor after the LignoBoost process increased slightly with the higher initial active alkali loads. In the kraft process, the amount of precipitated lignin ranged from 11 to 19% for sugar cane bagasse and 17–21% for sugar cane straw. In the soda process, these values were 11–17% and 14–19%, respectively. These lignin recovery yields are consistent with previous studies using kraft pulping liquors derived from wood feedstocks (Tomani, 2009; Bertaud et al., 2023).^15,16^ Higher lignin recovery yields have been reported under optimized precipitation conditions, including elevated temperatures (up to 120 °C) and CO_2_ pressurization (up to 5 bar), resulting in lignin precipitation yields as high as 85% (Yiamsawas et al., 2023).^27^

**Figure 2:**
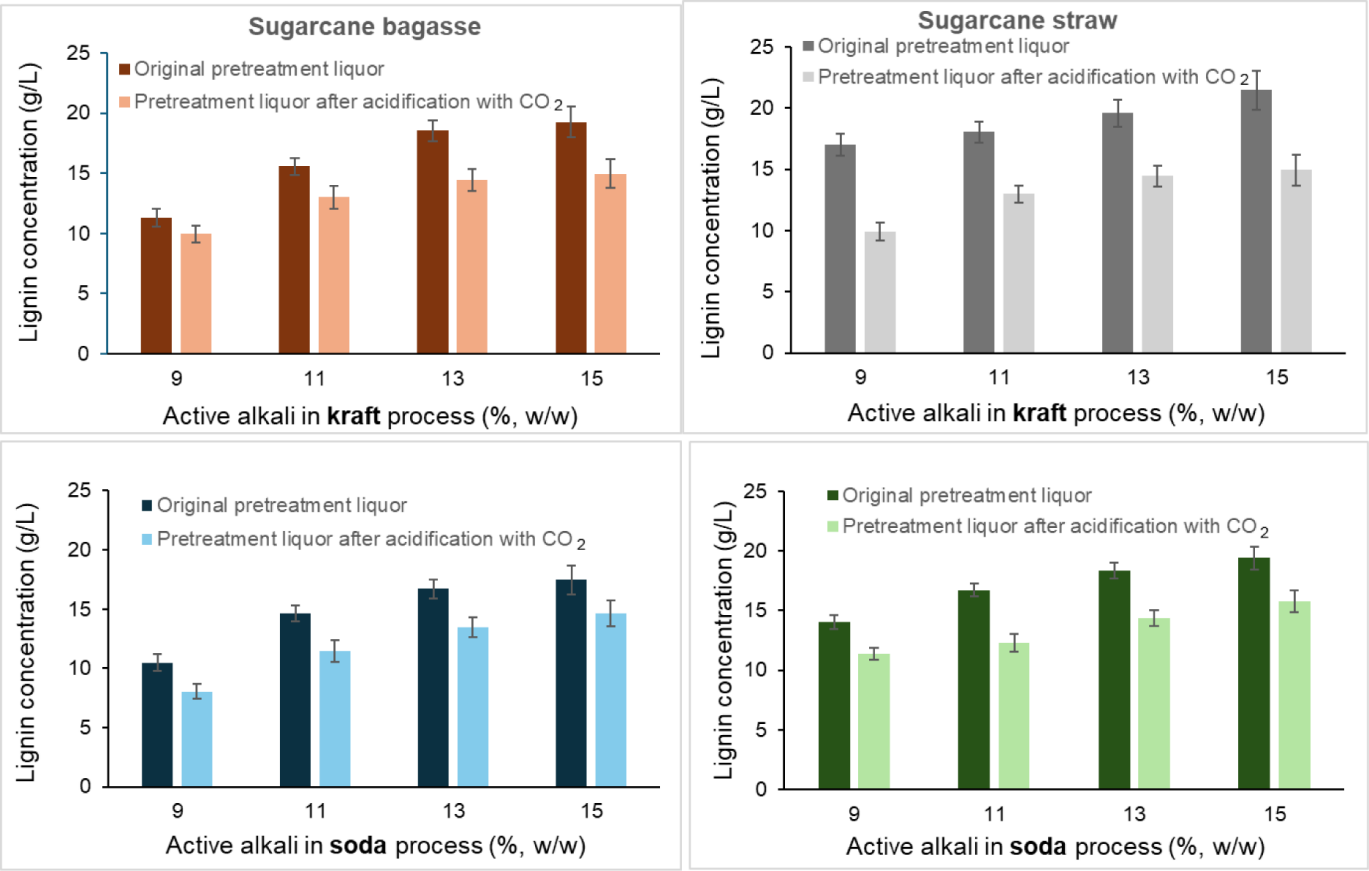
Lignin concentrations in the pretreatment liquors before and after lignin precipitation by a modified Lignoboost process. Pretreatment liquors were produced after kraft and soda pretreatments of sugar cane bagasse and straw under varied active alkali loads. In the kraft pretreatment, sulfidity was fixed at 25%.

Given that high monosaccharide yields were obtained under mild active alkali loads during the pretreatment step and that lignin recovery yields were comparable across all pretreatment liquors, subsequent experiments focused on using lignin recovered from the mildest condition (9% w/w active alkali) to reduce chemical consumption in the proposed biorefinery process.

### Ash Contents and Elemental Analysis from Lignin Samples

Straw-derived lignin exhibited high ash contents, reaching 18.4 ± 0.1% and 13.9 ± 0.2% in samples obtained from the kraft and soda processes, respectively. In contrast, bagasse lignin from the same processes contained significantly lower ash levels, at 5.6 ± 0.1% and 4.6 ± 0.1%, respectively. The elevated ash content in straw lignin is primarily attributed to the inherently high ash content of sugar cane straw feedstock (Table 1). However, contributions may also arise from adsorbed inorganic compounds introduced during the pretreatment and lignin precipitation steps. The contents of carbon (C), hydrogen (H), oxygen (O), and sulfur (S) are listed in Table 2. As expected, significant sulfur levels were detected only in the kraft lignin. High carbon content is particularly relevant as the lignin was subsequently subjected to pyrolysis for biochar production in this study. Kraft lignin exhibited slightly lower carbon contents, likely due to the presence of sulfur and relatively higher oxygen levels, in contrast to the soda lignin.

**Table 2:** Elemental Analysis of Sugarcane Bagasse and Straw Lignin Obtained through Kraft and Soda Pulping Processes Performed at 9% w/w Active Alkali^a^

bioresource/process	C	H	S	O
bagasse/kraft	59.7 ± 0.1	6.4 ± 0.1	1.9 ± 0.1	32.0 ± 0.3
straw/kraft	57.1 ± 0.1	7.1 ± 0.1	1.2 ± 0.1	34.6 ± 0.3
bagasse/soda	62.3 ± 0.1	6.9 ± 0.1		30.8 ± 0.2
straw/soda	62.4 ± 0.2	7.5 ± 0.2		30.2 ± 0.4

a Lignin was recovered after precipitation with CO_2_ using the Lignoboost process. Data are expressed based on ash-free samples.

### Pyrolysis Products from Lignin Samples

Py-GC/MS analysis of the samples provided valuable insights into the aromatic structure of the lignin through the identification of products of its fast pyrolytic degradation. In this study, we investigated the formation of the main phenolic compounds derived from H, G, and S units in lignin. In addition, attention was put on the presence of catechol (C) in the pyrolysis products since this compound can result from G and S units during secondary pyrolysis reactions at 400–450 °C (Kawamoto, 2017).^28^ Py-GC/MS chromatograms are shown in Figure 3, while a list of the main detected products is presented in Tables 3 and S3. The high resolution and sensitivity of the Py-GC/MS method enable the identification of numerous pyrolysis products, while the relative abundance of each compound is determined from its peak area relative to the total area of phenolic compounds.

**Figure 3:**
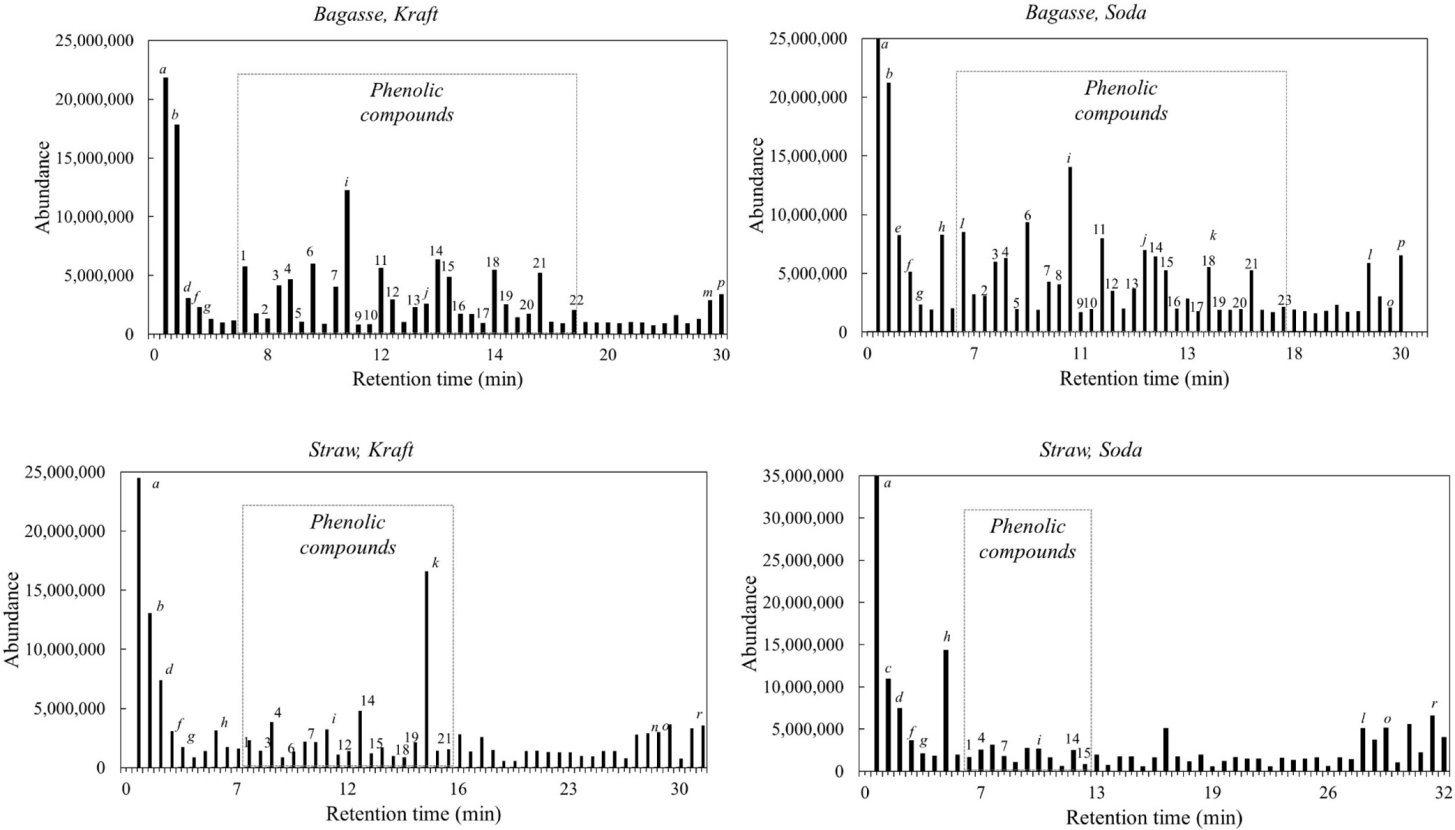
Comparison of Py-GC/MS total ion chromatograms of the sugar cane bagasse (top) and straw (bottom) lignin extracted using kraft (left) and soda (right) processes. The lignin-derived phenolic compounds have been assigned by numbers (1–23) and their retention time range have been marked by dotted lines, while other main detected products are indicated by small letters (a–r).

**Table 3:** Major Pyrolysis Products Identified in the Py-GC/MS Evaluation of the Lignin Samples Recovered from Sugarcane Bagasse and Straw^a^

peak name	phenolic compounds	unit type	peak name	other major compounds
1	phenol	H	a	carbon dioxide
2	phenol, 2-methyl-	H	b	methyl alcohol
3	*p*-cresol (phenol, 4-methyl-)	H	c	butyl alcohol
4	*o*-guaiacol(phenol, 2-methoxy-)	G	d	acetic acid, trifluoro-, 3-methylbutyl ester
5	phenol, 2,4-dimethyl-	H	e	furan, tetrahydro-3-methyl-4-methylene-
6	phenol, 4-ethyl-	H	f	1-hexene
7	phenol, 2-methoxy-4-methyl-	G	g	1-heptene
8	catechol (*o*-dihydroxybenzene)	C	h	furfural
9	*p*-cumenol (1-hydroxy-4-isopropylbenzene)	H	i	benzofuran, 2,3-dihydro-
10	phenol, 2-ethyl-5-methyl-	H	j	benzofuran, 2,3-dihydro-2-methyl-
11	pyrocatechol, 3-methoxy-	G	k	1,6-anhydro-.beta.-d-glucofuranose
12	phenol, 4-ethyl-2-methoxy-	G	l	tricosyl trifluoroacetate
13	(1,2-benzenediol, 4-methyl-)	C	m	octacosanol
14	2-methoxy-4-vinylphenol	G	n	octacosane, 2-methyl-
15	phenol, 2,6-dimethoxy-	S	o	octacosyl trifluoroacetate
16	phenol, 3,4-dimethoxy-	S	p	octacosanol
17	phenol, 2-methoxy-4-(1-propenyl)-	G	r	octacosyl trifluoroacetate
18	3,5-dimethoxy-4-hydroxytoluene	S		
19	phenol, 2-methoxy-4-(1-propenyl)-	G		
20	benzene, 1,2,3-trimethoxy-5-methyl-	S		
21	phenol, 4-ethenyl-2,6-dimethoxy-	S		
22	phenol, 2,6-dimethoxy-4-(1-propenyl)-	S		
23	phenol, 4-acetyl-2,6-dimethoxy	S		

a Peak names refer to the peaks identified in the GC/MS chromatogram described in Figure 3.

Lignin derived from sugar cane bagasse produced significantly higher total peak areas of phenolic compounds in the Py-GC/MS analysis (37–43%) compared to those from sugar cane straw (5–13%). The diversity of phenolic compounds formed was also greater in bagasse lignin than in straw lignin. The high ash content observed in straw lignin may have reduced the effective amount of lignin available for pyrolysis, as the Py-GC/MS experiments were conducted by using similar initial lignin masses. The detection of carbohydrate pyrolysis products (compounds h and k, Table 3) also suggests that the samples may contain polysaccharides, likely originating from cell wall debris that passed through the 20-mesh nylon screen used to separate pulps from pretreatment liquors, and subsequently precipitated during lignin recovery via the LignoBoost process.

The ratio of phenolic compounds derived from the H, G, and S units can provide an estimate of the structural composition of the studied lignin (Table 4). In grass lignin, *p*-coumarate esterified to lignin typically produces significant amounts of 4-vinylphenol during pyrolysis (Del Río et al., 2015).^29^ However, in the present Py-GC/MS experiments, 4-vinylphenol was not detected, likely because the *p*-coumarate moieties were saponified during the pretreatment step and remained in the liquor following lignin precipitation. In contrast, the Py-GC/MS data revealed significant levels of 2-methoxy-4-vinylphenol (compound 14, Table 3), a pyrolysis product of ferulate, which is commonly esterified to xylan in grasses. This finding also suggests the occurrence of polysaccharide contamination in the samples. The H/G/S ratio of the bagasse lignin recovered in this study showed a higher proportion of H units compared to milled wood lignin from the same substrate, as reported by Del Río et al. (2015).^29^ In the case of straw lignin, G units were predominant, consistent with previous findings for milled wood lignin from sugar cane straw, also reported by Del Río et al. (2015).^29^ Catechol, a potential precursor of polycyclic aromatic hydrocarbons (PAHs) (Wornat et al., 2001; Kawamoto, 2017),^28,30^ was detected only in the pyrolysis products of bagasse lignin. However, its relative concentration was low, with peak areas corresponding to 1% and 3% for the kraft and soda processes, respectively. No PAHs were detected among the pyrolysis products of any of the samples analyzed in this study. It is important to note, however, that PAH formation typically begins at around 600 °C (Kawamoto, 2017)^28^—the maximum temperature applied in our Py-GC/MS experiments—and is known to accelerate at temperatures above 700 °C (Kawamoto, 2017).^28^ Therefore, the formation of PAHs during the pyrolysis of bagasse lignin at higher temperatures cannot be ruled out.

**Table 4:** HGS Structural Subunits in Technical Lignin Recovered from Sugarcane Bagasse and Straw Samples Estimated Based on Py-GC/MS Phenolic Products

lignin structural type	% based on the Py-GC/MS area of the corresponding phenols			
	bagasse		straw	
	kraft	soda	kraft	soda
H	29	37	19	21
G	40	37	63	68
S	31	25	18	11

Other studies on the pyrolysis and copyrolysis of lignin with other carbon-rich materials have been reported (Chen et al., 2019; Chen et al., 2024; del Río et al., 2015; Haz et al., 2013; Kawamoto, 2017; Mullen and Boateng, 2010; Wang et al., 2023).^28,29,31–35^ However, a general comparison of lignin pyrolysis products obtained under different experimental conditions is challenging, as each Py-GC/MS analysis is strongly influenced by the type of lignin feedstock, feedstock pretreatment, and the applied pyrolysis parameters (e.g., temperature, heating rate, residence time, and catalysts) (Chen et al., 2019; Chen et al., 2024; del Río et al., 2015; Haz et al., 2013; Kawamoto, 2017; Mullen and Boateng, 2010; Wang et al., 2023).^28,29,31–35^

### Biochar Production and Characterization

Pyrolysis is considered a simple but practical method for efficient conversion of lignin into valuable biomaterials, biofuels, and biochemicals (Chen et al., 2019; Chen et al., 2024; Kawamoto, 2017; Mullen and Boateng, 2010; Wang et al., 2023).^28,31,32,34,35^ The lignin conversion is a complex process with different simultaneous reactions and cross-linked mechanisms (Kawamoto, 2017).^26^ In this study, we focused exclusively on the characterization of the final biochar product generated during the pyrolysis of different lignin samples derived from sugar cane at the same pyrolytic conditions.

A three-step pyrolytic conversion of lignin applied in this study produced biochar in a yield of 36–43% of the initial mass of raw material (Table 5). The biochar yield was slightly higher in the case of lignin obtained from sugar cane bagasse (36–43%) than straw (36–40%), which may be related to a higher amount of ash detected in the latter material (Table 1). Moreover, considering the lignin materials originated from the same source but extracted using different processes, it seems that the kraft process favors biochar formation over the soda process by 6.8% and 3.8% for bagasse and straw, respectively (Table 5).

**Table 5:** Conversion Yields of Lignin Recovered from Sugarcane Bagasse and Straw to the Corresponding Biochars

lignin source	extraction process	lignin before pyrolysis (mg)	biochar after pyrolysis at 1000 °C (mg)	biochar yield (%)
bagasse	kraft	1.222	0.527	43.1
	soda	1.412	0.512	36.3
straw	kraft	1.143	0.454	39.7
	soda	1.359	0.488	35.9

#### Structural and Chemical Characterization of Biochar Samples

The structure and chemical composition of biochar samples were analyzed by using SEM–EDX and Raman spectroscopy. Figure 4 shows SEM images of lignin-derived biochar samples. All obtained biochar materials were characterized by the presence of layered plates decorated with a grainy material. The amount of the grainy material and porosity was higher on the surface of biochar derived from straw (c,d), reflecting higher ash content found in lignin samples derived from straw.

**Figure 4:**
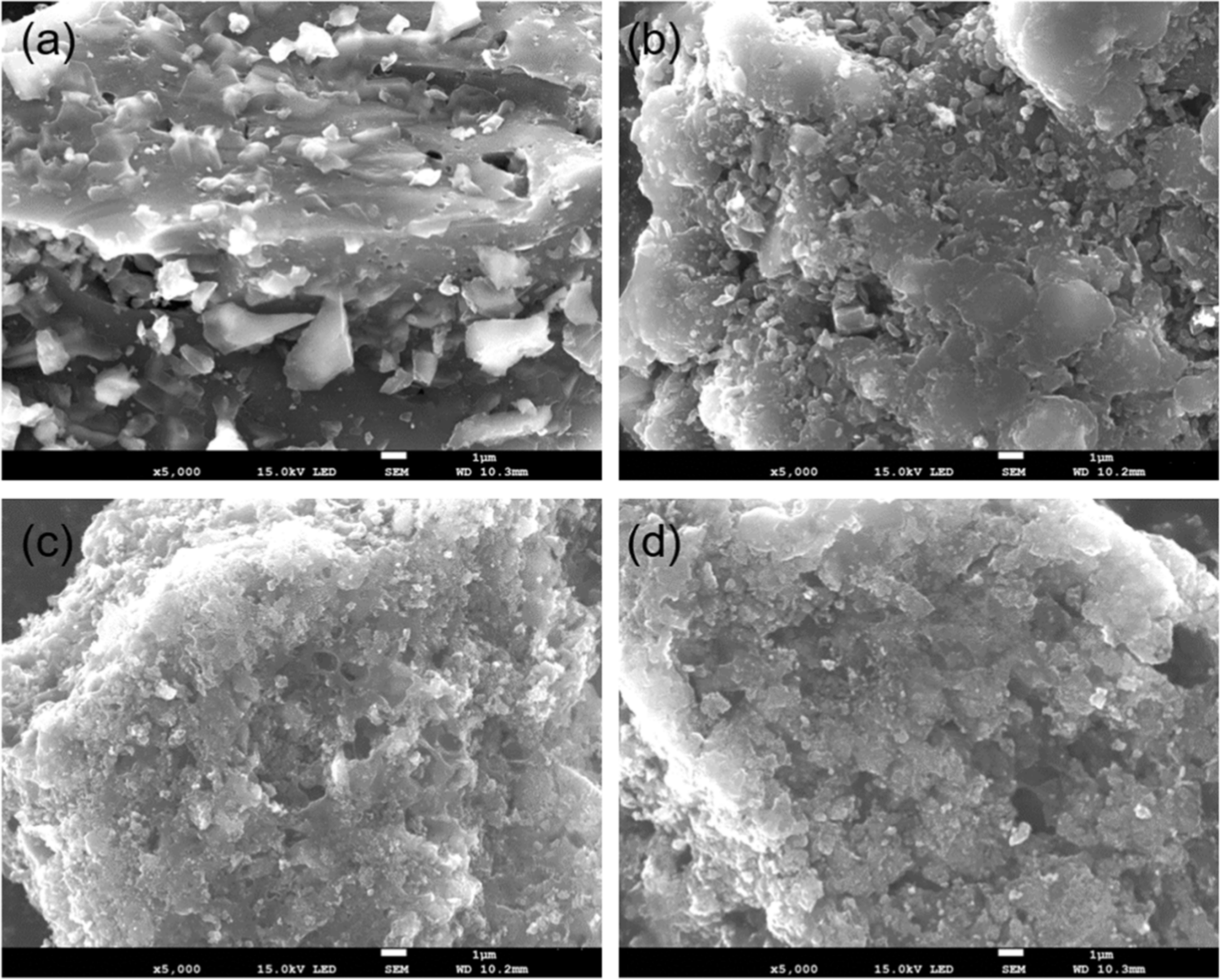
SEM images of biochar samples derived from sugar cane bagasse pretreated by modified kraft pulping (a) and soda pulping processes (b). (c,d) refer to sugar cane straw lignin from the kraft and soda processes, respectively.

Elemental analysis by EDX revealed significant differences in the chemical compositions of biochar samples (Figure 5). Particularly, straw-derived biochar samples contained higher amounts of silicon than bagasse biochars. On the other hand, as expected from the nature of the extraction process, biochars derived from lignin using kraft pretreatment contained a higher amount of sulfur. These differences in the elemental composition have a significant impact on the electrochemical performance of biochars as discussed later.

**Figure 5:**
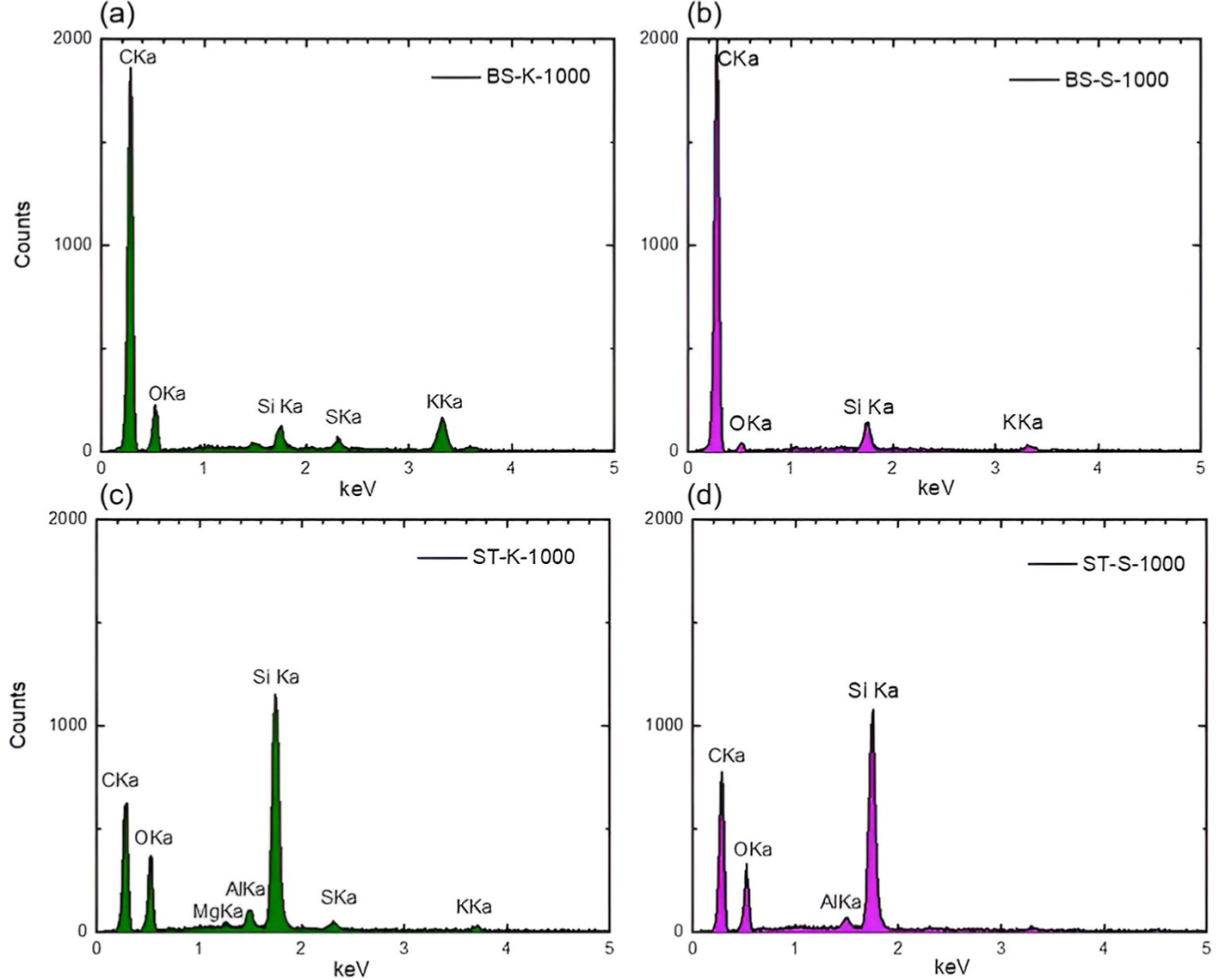
EDX spectra of biochar samples derived from sugar cane bagasse biochar pretreated by kraft (a) and soda processes (b). (c,d) refer to sugar cane straw biochar from the kraft and soda processes, respectively.

Raman spectroscopy was used to assess the degree of graphitization and the quality of the biochars. Figure 6 shows Raman spectra for all biochar samples. Spectra were characterized by two main peaks, one at 1351 and the other at 1592 cm^–1^ corresponding to disorder graphitic (D), and graphitic (G) carbon, respectively. In comparison to commercial carbons derived from the thermal decomposition of hydrocarbons such as acetylene black, the D and G bands of lignin biochars are broader, indicating the presence of other structural forms such as amorphous carbon, graphene, or graphene edges (Sadezky et al., 2005).^36^

**Figure 6:**
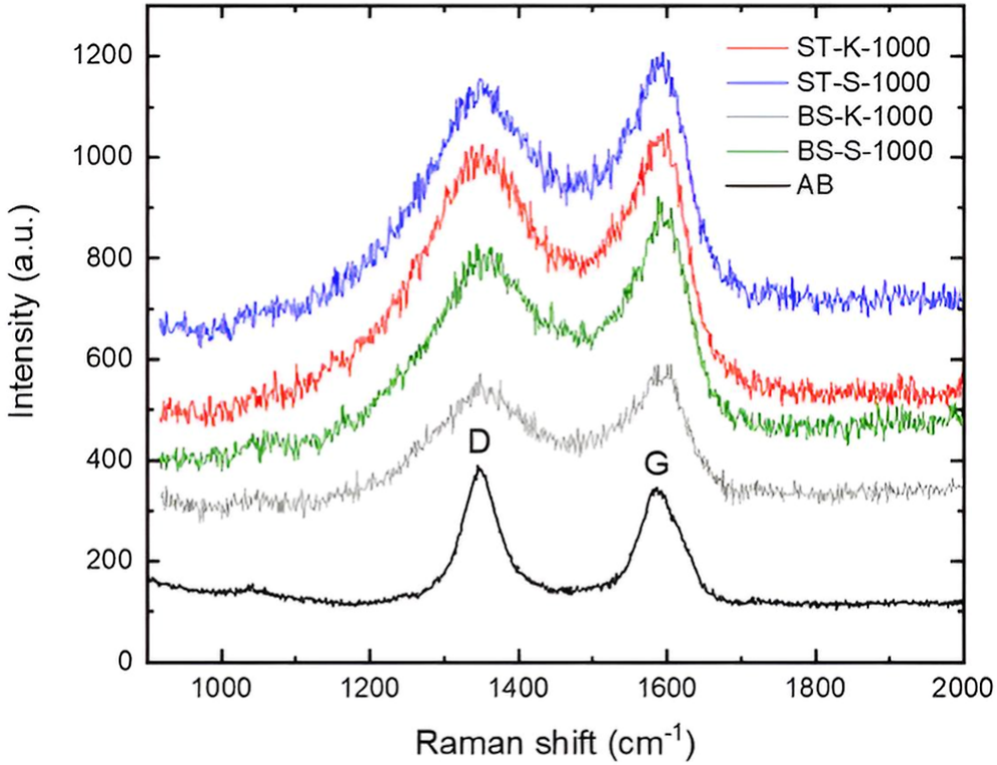
Raman spectra of lignin-derived biochar samples recovered from sugar cane bagasse and straw are compared with commercial acetylene black (AB). In the sample labels, ST and BS indicate straw- and bagasse-derived biochars, respectively, while K and S denote kraft and soda pretreatments. AB represents the acetylene black reference material.

#### Electrochemical Tests

Biochar samples were evaluated for their electrochemical behavior in alkaline media, highlighting their potential for both catalytic OER/oxygen reduction reaction (ORR) and insertion-type battery applications (Putra et al., 2022 ^19^). Figure 7 presents the LSV curves in the oxidative potential range of 0.9–1.5 V in 1 M KOH. For bagasse-derived biochars, the oxidative currents were relatively low, reaching 0.07 mA cm^–2^ at 1.56 V (Figure 7a), with an onset of electrooxidation around 1 V. Initially, the rate of current increase was slightly higher for bagasse kraft than for bagasse soda biochar electrodes, but above 1.4 V, the kraft-derived biochars showed a more pronounced increase, indicating slightly lower electrochemical stability.

**Figure 7:**
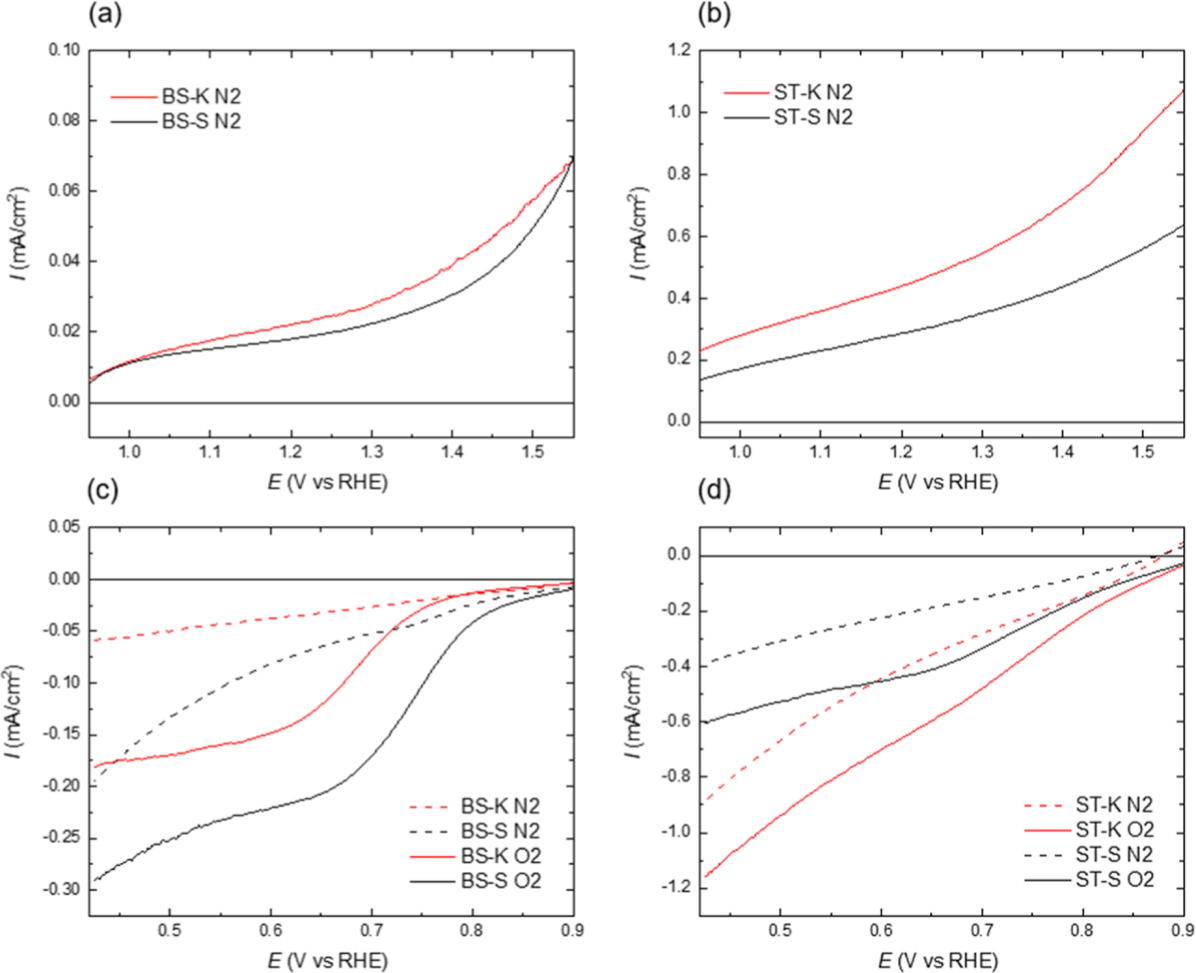
LSV curves for (a) bagasse, kraft (K) and soda (S) samples, and (b) straw, kraft (K) and soda (S) samples in the OER potential window; (c,d) correspond to the same samples in the ORR potential window.

In contrast, straw-derived biochar electrodes exhibited oxidative currents approximately ten times higher than those of bagasse biochars. This enhanced current may seem counterintuitive as straw biochars contain a higher content of silicon, likely in the form of magnesium aluminum silicates, which are poor electrical conductors. Despite the slower depolarization expected from silicate-rich materials, their higher overall conductivity suggests potential utility as conductive additives in insertion-type battery electrodes, even if their stability as catalytic OER carbons is limited under these conditions.

The lower potential region (Figure 7c,d) was analyzed to assess the ORR activity. LSV measurements were conducted in both nitrogen- and oxygen-saturated KOH solutions, with dotted lines representing N_2_-saturated and solid lines representing the corresponding O_2_-saturated electrolytes. For bagasse biochars, small ORR currents were observed, with onset potentials of 0.82 V for soda-derived and 0.76 V for kraft-derived samples, indicating modest catalytic activity. Straw-derived biochars, however, showed no defined ORR peaks, with currents increasing monotonically with applied potential, reflecting their limited catalytic suitability but reinforcing their role as conductive materials for battery applications.

## Conclusions

Delignification of sugar cane bagasse and straw was effective using both kraft and soda processes. The kraft process achieved more extensive delignification, yielding cellulose pulps with a lower residual lignin content under all tested conditions. The pretreated pulps were readily hydrolyzed by cellulases, producing high monosaccharide yields, even under mild alkali pretreatment conditions. Lignins recovered from the pretreatment liquors were identified as the HGS type via Py-GC/MS analysis. Pyrolysis of the recovered lignins produced biochars with yields ranging from 36% to 43%. Compositional analysis revealed that straw-derived biochars contained higher silicon content, whereas kraft-derived biochars were enriched in sulfur.

The chemical composition of lignin-derived biochars influenced their electrochemical behavior in alkaline media. Electrodes prepared from bagasse biochars exhibited modest ORR currents, with onset potentials between 0.76 and 0.82 V, indicating limited catalytic activity for oxygen reduction and evolution reactions. In contrast, straw-derived biochars, containing higher silica levels, showed pronounced electrochemical instability at both positive and ORR potentials, restricting their suitability as catalytic carbons in energy conversion devices. Nevertheless, the higher conductivity observed in straw biochars suggests potential utility as conductive additives in insertion-type battery electrodes, highlighting the application-dependent performance of these lignin-derived materials. These findings demonstrate that the electrochemical functionality of biochars can be tuned by the biomass source and pretreatment, enabling dual roles as catalytic or conductive components depending on the targeted energy storage and conversion application.

## Supplementary Material



## Data Availability

Data are contained within the manuscript and in the Supporting Information as well.

## Notes

Data on lignin recovery under all delignification conditions, lignin yield calculations (L/L), pH values, and lignin concentrations obtained before and after pretreatment; and pyrolysis products of the lignin samples as identified by Py-GC/MS analysis (PDF)
