# Supplementary material for: Upcycling Sugar Cane Biomass into 2G Sugars and Lignin-Derived Biochars for Preparing Carbon-Based Electrodes
Source: ACS Omega. 2025 Oct 30;10(44):53378–89. doi: 10.1021/acsomega.5c08482 (PMC12612950; doi:10.1021/acsomega.5c08482)
Supplement: Supplementary file 1 [file ao5c08482_si_001.pdf]

Upcycling Sugarcane Biomass into 2G Sugars and Lignin Derived Biochars for Preparing Carbon-based Electrodes

Lucas Ramos[1], Talita M. Lacerda[1], André Ferraz[1], Mariusz Grabda[2], Sylwia Oleszek[3], Hideyuki Horino[4], Izabela Rzeznicka[5*], Anuj Kumar Chandel[1*]

1 Renewable Carbon and Biology Systems (ReCABS) Laboratory, Department of Biotechnology, Engineering School of Lorena, University of São Paulo (EEL-USP), Lorena-12602-810, SP, Brazil

2 Shibaura Institute of Technology Research Laboratories, Saitama 337-8570, Japan

3 Department of Environmental Engineering, Graduate School of Engineering, Kyoto University, Kyoto 615-8540, Japan

4 Research Management Center, Tohoku University, Sendai 982-8577, Japan

5 College of Engineering, Shibaura Institute of Technology, Saitama 337-8570, Japan

* Correspondence: anuj10@usp.br; anuj.kumar.chandel@gmail.com

Table S1. Pretreatment liquor characteristics resulting from sugarcane bagasse and straw submitted to kraft pulping pretreatment

	**Active álcali (%)**	**Yield (L/L)**	**Liquor pH**	**Lignin concentration (g/L) before preciptation**	**Liquor pH after CO_2 flux**	**Lignin Concentration (g/L) after preciptation**
Bagasse	9%	0.92	8.2	11.3	7.07	9.9
	11%	0.92	9.28	15.1	7.62	13.0
	13%	0.83	9.68	18.5	7.85	14.5
	15%	0.83	10.14	27.5	8.52	14.9
Straw	9%	0.75	7.88	17.0	6.88	13.0
	11%	0.76	8.44	18.0	7.35	13.2
	13%	0.78	9.26	19.6	7.60	16.5
	15%	0.9	9.76	21.5	7.85	17.1

Table S2. Pretreatment liquor characteristics resulting from sugarcane bagasse and straw pretreatment under soda pulping

	**Active álcali (%)**	**Yield (L/L)**	**Liquor pH**	**Lignin concentration (g/L) before preciptation**	**Liquor pH after CO_2 flux**	**Lignin concentration(g/L) after preciptation**
Bagasse	9%	0.77	8.62	10.5	6.7	8.1
	11%	0.79	9.3	14.6	7.02	11.5
	13%	0.77	9.6	16.6	7.10	13.5
	15%	0.77	10.17	25.2	7.26	14.7
Straw	9%	0.79	7.82	14.1	6.65	11.4
	11%	0.78	8.31	16.7	6.83	12.3
	13%	0.79	8.83	18.4	7.10	14.4
	15%	0.79	9.85	19.4	7.25	15.8

Table. S3. Py-GC/MS analysis of all volatile products in the pyrolysis of the sugarcane bagasse and straw lignin extracted using the kraft and soda process

RT (min)	Peak name	Product name	MW	Unit type	***Bagasse***		***Straw***		CAS No.
					Kraft	*Soda*	*Kraft*	*Soda*	
1.554<br>1.56<br>1.572	*a*	Carbon dioxide	44		-<br>13.57	10.71	16.19	-<br>20.92	124-38-9
1.616	*b*	Methyl Alcohol	32		11.08	8.97	8.65	-	67-56-1
1.62	*c*	Butyl Alcohol	74		-	-	-	6.53	71-36-3
1.744<br>1.747	*d*	Acetic acid, trifluoro-, 3-methylbutyl ester	184		1.91<br>-	-<br>-	4.87<br>-	-<br>4.45	327-69-5
1.754	*e*	Furan, tetrahydro-3-methyl-4-methylene-	98		-	3.49	-	-	61142-01-6
2.018<br>2.023	*f*	1-Hexene	84		1.43<br>-	-<br>2.19	-<br>2.05	-<br>2.18	592-41-6
2.664	*g*	1-Heptene	98		0.82	1	1.15	1.29	592-76-7
3.562		Benzene, methyl-	92		-	-	0.58	-	108-88-3
3.892		1-Octene	112		0.63	0.82	0.92	1.13	111-66-0
4.537	*h*	Furfural	96		-	3.51	2.09	8.52	98-01-1
5.574		1-Nonene	126		0.73	0.87	1.16	1.18	124-11-8
7.235	*1*	Phenol	94	H	3.59	3.61	1.05	1.02	108-95-2
7.342		1-Decene	140		1.11	1.36	1.54	1.54	872-05-9
8.466	2	Phenol, 2-methyl-	108	H	0.84	1.3	-	-	95-48-7
8.821	3	p-Cresol (Phenol, 4-methyl-)	108	H	2.58	2.54	0.96	-	106-44-5
9.035<br>9.041	4	o-Guaiacol (Phenol, 2-methoxy-)	164	G	2.91<br>-	-<br>2.67	-<br>2.53	-<br>1.07	90-05-1
9.049		1-Dodecene	168		-	-	-	1.87	112-41-4
9.985	5	Phenol, 2,4-dimethyl-	122	H	0.67	0.83	-	-	105-67-9
10.28	6	Phenol, 4-ethyl-	122	H	3.74	3.97	0.57	-	123-07-9
10.628		1-Dodecene	168		0.57	0.8	0.91	-	112-41-4
10.682	7	Phenol, 2-methoxy-4-methyl-	138	G	2.50	1.83	1.45	0.68	93-51-6
10.857	8	Catechol (o-Dihydroxybenzene)	110	C	-	1.73	-	-	120-80-9
10.884		1,4:3,6-Dianhydro-.alpha.-d-glucopyranose	144		-	-	1.43	1.64	0-00-0
11.074	*i*	Benzofuran, 2,3-dihydro-	120		7.62	5.94	2.14	1.58	496-16-2
11.184	9	p-Cumenol (1-Hydroxy-4-isopropylbenzene)	136	H	0.50	0.73	-	-	99-89-8
11.349	10	Phenol, 2-ethyl-5-methyl-	136	H	0.53	0.83	-	-	1687-61-2

11.751	11	Pyrocatechol, 3-methoxy-(1,2-Benzenediol, 3-methoxy-)	140	G	3.49	3.38	-	-	934-00-9
11.966	12	Phenol, 4-ethyl-2-methoxy-	152	G	1.84	1.48	0.73	-	2785-89-9
12.102<br>12.108<br>12.159		1-Tridecene	182		0.65<br>-<br>-	0.86<br>-<br>-	0.94<br>-<br>-	-<br>0.97<br>0.38	2437-56-1
12.19	13	4-Methylcatechol (1,2-Benzenediol, 4-methyl-)	124	C	1.42	1.56	-	-	452-86-8
12.29	*j*	Benzofuran, 2,3-dihydro-2-methyl-	134		1.61	2.95	-	-	1746-11-8
12.476	14	2-Methoxy-4-vinylphenol	150	G	3.97	2.73	3.18	1.5	7786-61-0
12.97	15	Phenol, 2,6-dimethoxy-	154	S	3.04	2.23	0.8	0.52	91-10-1
13.13	16	Phenol, 3,4-dimethoxy-	154	S	1.09	0.85	-	-	2033-89-8
13.486<br>13.489		1-Tridecene	182		-<br>1.06	1.21<br>-	1.12<br>-	1.17<br>-	2437-56-1
13.594	17	Phenol, 2-methoxy-4-(1-propenyl)-	164	G	0.60	0.76	-	-	97-54-1
13.599		Octane, 2,3,3-trimethyl	156		-	-	-	0.43	62016-30-2
14.247	18	3,5-Dimethoxy-4-hydroxytoluene	168	S	3.42	2.33	0.66	-	07/05/6638
14.325	19	Phenol, 2-methoxy-4-(1-propenyl)-	164	G	1.58	0.8	0.56	-	97-54-1
14.787<br>14.788<br>14.792		1-Pentadecene	210		-<br>0.89	-<br>0.81	1.4<br>-	-<br>-<br>1.04	13360-61-7
15.251	20	Benzene, 1,2,3-trimethoxy-5-methyl-	182	S	1.08	0.83	-	-	6443-69-2
15.258	*k*	1,6-Anhydro-.beta.-D-glucofuranose	162		-	-	10.98	-	7425-74-3
15.746	21	Phenol, 4-ethenyl-2,6-dimethoxy-	180	S	3.24	2.23	0.95	-	28343-22-8
16.015<br>16.016		Cetene	224		-<br>0.68	0.8<br>-	-<br>-	-<br>1.06	629-73-2
16.115		Tetradecane	198		-	-	1.03	0.34	629-59-4
16.301		1,6-Anhydro-.beta.-D-glucofuranose	162		-	-	1.87	-	7425-74-3
17.18<br>17.185		Cetene	224		0.57<br>-	0.71<br>-	0.91<br>-	0.99	629-73-2
17.334	22	Phenol, 2,6-dimethoxy-4-(1-propenyl)-	194	S	1.29	-	-	-	20675-95-0
17.611		1-Dodecanol, 3,7,11-trimethyl-	228		-	-	1.7	-	6750-34-1
17.618		Cycloundecane, 1,1,2-trimethyl-	112		-	-	-	3.04	62376-15-2
17.724	23	Phenol, 4-acetyl-2,6-dimethoxy	196	S	-	0.91	-	-	2478-38-8

18.284<br>18.285		9-Eicosene, (E)-	281		0.65<br>-	0.82<br>-	-<br>0.97	1.05<br>-	74685-29-3
18.788<br>19.237		Neophytadiene	279		-<br>-	-<br>-	0.38<br>0.36	0.69<br>-	504-96-1
19.336		9-Eicosene, (E)-	281		-	0.76	-	-	74685-29-3
19.338		3-Eicosene, (E)-	281		-	-	0.94	-	74685-33-9
19.339		1-Heptadecene	238		0.62	-	-	-	6765-39-5
19.343		1-Octadecene	252		-	-	-	1.17	112-88-9
19.415		Hexadecane	226		-	-	-	0.36	544-76-3
19.485		2-Tridecylfuran	250		-	-	-	0.74	25346-22-9
20.342<br>21.301		1-Octadecene	252		0.62	0.69	0.96<br>0.87	1<br>0.9	112-88-9
21.302		1-Heptadecene	239		0.57	-	-	-	6765-39-5
22.221		Trifluoroacetoxy hexadecane	338		0.64	-	-	-	03/03/6222
22.221		1-Hexacosene	365		-	-	0.84	-	18835-33-1
22.226		1-Octadecene	252		-	-	-	0.9	112-88-9
22.276		Heptadecane	240		-	-	-	0.36	629-78-7
23.102<br>23.948<br>24.761		1-Hexacosene	365		0.62<br>0.49<br>0.59	-<br>-<br>0.77	0.85<br>0.64<br>0.62	0.96<br>0.79<br>0.9	18835-33-1
25.544		Behenic alcohol	327		-	0.97	-	-	661-19-8
25.545		1-Nonadecene	267		-	-	0.91	-	18435-45-5
25.546		1-Hexacosene	365		1.03	-	-	0.98	18835-33-1
25.585		Eicosane, 1-iodo-	408		-	-	-	0.39	0-00-0
26.3		Behenic alcohol	327		0.58	0.73	-	-	661-19-8
26.301		1-Nonadecene	267		-	-	0.93	-	18435-45-5
26.307		n-Tetracosanol-1	355		-	-	-	0.97	506-51-4
26.335		Heneicosane	297		0.82	0.76	0.53	-	629-94-7
26.34		Octadecane, 1-iodo-	380		-	-	-	0.84	629-93-6
27.03	*l*	Tricosyl trifluoroacetate	437		-	2.48	-	3.03	0-00-0
27.031	*m*	Octacosanol	380		1.81	-	-	-	557-61-9
27.032		Heneicosyl heptafluorobutyrate	509		-	-	1.82	-	0-00-0
27.798	*n*	Octacosane, 2-methyl-	409		-	-	1.9	-	1560-98-1
27.799		Pentatriacontane	493		-	1.29	-	-	630-07-9
27.804		Tetratetracontane	619		-	-	-	2.21	7098-22-8
28.578	*o*	Octacosyl trifluoroacetate	507		-	0.88	1.98	3.07	0-00-0
29.338		Stigmasta-5,22-dien-3-ol, acetate, (3.beta.)-	455		-	-	-	0.63	4651-48-3
29.508		Heneicosane	297		-	-	2.4	-	629-94-7
29.514		Tetratetracontane	619		-	-	-	3.33	7098-22-8
29.565		n-Nonadecanol-1	285		-	-	0.51	-	1454-84-8
29.566	*p*	Octacosanol	411		2.12	2.76	-	-	557-61-9
29.912		Stigmast-5-en-3-ol, acetate, (3.beta.)-	457		-	-	-	1.33	915-05-9
30.528	*r*	Octacosyl trifluoroacetate	507		-	-	2.19	3.93	0-00-0
31.787		Tetratetracontane	619		-	-	2.36	-	7098-22-8
31.798		1-Pentacosene	350		-	-	-	2.42	16980-85-1
